# Previsit Preparation for Shared Decision-Making in Lung Cancer Screening in Primary Care Using a Paper Decision Aid and an Automated Text Messaging Program: Quasi-Experimental Pilot Study

**DOI:** 10.2196/69044

**Published:** 2025-09-17

**Authors:** Mayuko Ito Fukunaga, Renda Soylemez Wiener, Shaun Toomey, Joann Wagner, Qiming Shi, Kavitha Balakrishnan, Alexandra Nguyen, Dan Nguyen, M Diane McKee, Alexander A Bankier, Rajani S Sadasivam, Sybil L Crawford, Paul KJ Han, Thomas K Houston, Kathleen M Mazor

**Affiliations:** 1 Department of Medicine, University of Massachusetts Chan Medical School, Worcester, MA, United States; 2 Center for Health Optimization and Implementation Research, VA Boston Healthcare System, Boston, MA, United States; 3 National Center for Lung Cancer Screening, Veterans Health Administration, Washington, DC, United States; 4 Pulmonary Center, Boston University Chobanian & Avedisian School of Medicine, Boston, MA, United States; 5 Center for Clinical and Translational Science, University of Massachusetts Chan Medical School, Worcester, MA, United States; 6 Department of Population and Quantitative Health Sciences, University of Massachusetts Chan Medical School, Worcester, MA, United States; 7 Department of Obstetrics and Gynecology, University of Arizona, Tucson, AZ, United States; 8 University of Massachusetts Chan Medical School, Worcester, MA, United States; 9 Department of Family Medicine and Community Health, University of Massachusetts Chan Medical School, Worcester, MA, United States; 10 Department of Radiology, University of Massachusetts Chan Medical School, Worcester, MA, United States; 11 Tan Chingfen Graduate School of Nursing, University of Massachusetts Chan Medical School, Worcester, MA, United States; 12 Center for Outcomes Research and Evaluation, Maine Medical Center, Portland, ME, United States; 13 Tufts University School of Medicine, Boston, MA, United States; 14 Division of Cancer Control and Population Sciences, National Cancer Institute, Bethesda, MD, United States; 15 Department of Internal Medicine, Wake Forest University School of Medicine, Winston-Salem, NC, United States

**Keywords:** text messaging, decision-making, shared, lung cancer, cancer screening, telemedicine, health

## Abstract

**Background:**

Patient-provider discussions and shared decision-making (SDM) are essential for tailoring lung cancer screening (LCS) decisions to individual patients. However, the implementation of SDM in primary care settings is challenging. Innovative approaches are needed to reach and prepare patients eligible for LCS for SDM in primary care settings and increase LCS uptake.

**Objective:**

We piloted previsit preparation comparing 2 strategies: a paper decision aid (DA; DA group) and an enhanced comparator strategy consisting of the paper DA plus an automated text message program (DA+TM group) designed to promote patient-provider LCS discussions. We explored feasibility and gathered preliminary data on its potential effects on LCS discussions, decision-making, and LCS uptake in primary care settings.

**Methods:**

In a sequential quasi-experimental pilot study, we recruited patients who were eligible for LCS in a single academic health care system. Prior to an upcoming visit, participants in both groups received a paper-based DA by mail. In the DA+TM group, participants also received a series of automated text messages to help them prepare for their LCS discussions. We monitored participant recruitment and retention, as well as patient engagement in DA and text messages. In exploratory analyses, we assessed patient-provider discussion of LCS, SDM, patient knowledge, decision conflict at baseline and in follow-up telephone surveys, and LCS completion measured by electronic health records.

**Results:**

We enrolled and included 48 participants (DA group=19 and DA+TM group=29) in the final analysis. Participants were predominantly White, with a median age of 61.0 (IQR, 57.0‐65.0), and 58% (28/48) of them were female. Engagement was high in both groups. LCS knowledge significantly improved in the DA+TM group (4.5 baseline vs 6.0 follow-up; *P*=.003), while there was no change in the DA group (5.0 baseline vs 5.0 follow-up, *P*=.23). Median LCS knowledge change from baseline to follow-up was 0.5 (IQR –1.0 to 2.5) in the DA group and 1.5 (IQR 0‐3.0) in the DA+TM group (*P*=.24). Decision conflict in both groups significantly decreased (DA group: 37.5 baseline vs 0 follow-up, *P*<.001; DA+TM group: 50.0 baseline vs 20.0 follow-up, *P*=.003). The median SDM process score (a measure of SDM) was 3.0 (IQR 1.5-4.0) in the DA group and 2.0 (IQR 1.0-3.0) in the DA+TM group (*P*=.11). The LCS completion rates were 5% (1/19) in the DA group and 31% (9/29) in the DA+TM group at 3 months (*P*=.07), and 26% (5/19) in the DA group and 34% (10/29) in the DA+TM group at 6 months (*P=*.75).

**Conclusions:**

Previsit preparation was feasible in primary care settings. An enhanced, text message–based strategy has the potential to reach and engage broader LCS-eligible populations and prepare patients for LCS discussions with their primary care providers, which may ultimately improve informed decision-making and LCS uptake.

## Introduction

Shared decision-making (SDM) is an essential component of lung cancer screening (LCS) with annual low-dose computed tomography (LDCT). Many medical professional societies, such as the US Preventive Services Task Force, recommend and the Centers for Medicare and Medicaid Services (CMS) has mandated SDM between patients and providers using a decision aid (DA) before enrolling in LCS. SDM is a process in which the patient and health care provider collaboratively review the best available evidence, consider the patient’s values and preferences, and then identify the most suitable option for the patient [1,2]. While LCS reduces lung cancer mortality, it also carries potential harms (eg, false positives, downstream invasive procedures and complications, radiation exposure, and anxiety). Furthermore, the balance of benefits and harms varies depending on an individual’s risks for lung cancer and comorbidities; thus, SDM is essential.

However, high-quality SDM for LCS rarely happens during clinical visits [3-8]. LCS is still relatively new compared to other cancer screenings. Many patients are not aware of LCS, including the screening process and its potential harms. Providers often discuss the potential benefits but not the potential harms of LCS, and few providers use DAs [3-8]. Due to multiple competing priorities, providers and patients often lack sufficient time to review LCS in depth and fully engage in SDM during primary care visits. [3,7]. Despite good intentions, the requirement for SDM for LCS is considered to be a barrier to LCS. Thus, an intervention to support and streamline SDM discussions is urgently needed.

One strategy to overcome some of the posed challenges is previsit preparation to introduce patients to key LCS facts and allow time for patients to prepare for SDM. By preparing patients, this strategy may also reduce the provider’s work to go over the information during the visit. Various DAs for LCS were developed, which improved patient knowledge and decreased decisional conflict in study settings [9,10]. Text messaging has been shown to increase patient knowledge and uptake of screenings for breast, cervical, and colon cancer and holds significant potential for effectively reaching and engaging the broader populations eligible for LCS. [11-13]

Thus, we developed two previsit preparation strategies of different intensities: (1) a mailed paper-based DA and (2) a mailed paper-based DA paired with a previsit automated text messaging program to support SDM for LCS. We then conducted a pilot study to test the feasibility of the 2 strategies and explore their potential effects on LCS discussions, decision-making, and LCS uptake at 3 and 6 months.

## Methods

### Study Design

We conducted a sequential quasi-experimental pilot study of previsit preparation using a paper-based DA (DA group) or the combination of paper-based DA and an automated text messaging program (DA+TM group). This study was conducted in accordance with CONSORT (Consolidated Standards of Reporting Trials) guidelines (Checklist 1).

### Study Setting and Participants

This study was conducted at 6 primary care clinics within a large academic health care system in the Northeastern United States from November 2020 to December 2022, during the COVID-19 pandemic. This health care system has an alert in the EPIC (Epic Systems Corporation) electronic health record (EHR) health maintenance section to inform providers of their patient’s potential eligibility for LCS based on age and smoking history. Primary care providers (PCPs) are encouraged to review the health maintenance section during visits.

Participants in this study were individuals eligible for LCS who never had an LCS before and who had an upcoming primary care visit. The inclusion criteria were (1) age 55-77 years, (2) cigarette smoking within the past 15 years with a minimum of 30 pack-years (ie, the 2015 CMS eligibility criteria for LCS) [14,15], (3) English-speaking, (4) owning a cell phone with text messaging capability, and (5) having an upcoming scheduled primary care visit. The exclusion criteria were (1) PCP’s exclusion of the patient based on their medical conditions (eg, under hospice care and severe mental illness), (2) active cancer, (3) history of lung cancer, (4) previous LCS, and (5) chest CT within the past 12 months.

### Recruitment Procedures

We identified patients potentially eligible for LCS who had an upcoming primary care appointment based on their age, histories of tobacco use, and previous chest imaging studies via EHR data. We recruited patients who had a primary care visit within 1‐4 weeks for the DA group and within 2‐5 weeks for the DA+TM group to ensure that the participants had enough time to receive the interventions prior to their visits and minimize the difference in the duration between the last day of the interventions (the day when the participants received DA in the DA group and the day when the participants received the last text message in the DA+TM group) and their scheduled visit days between the 2 groups.

We mailed invitation letters to all potentially eligible patients. One week after the mailing, a research staff member made a telephone call to those who did not opt out and confirmed study eligibility.

### Interventions

We delivered the paper-based DA developed by the Agency for Healthcare Research and Quality, which was publicly available during the study period. We developed an LCS text message program based on existing DAs in collaboration with patients eligible for LCS [16]. The text message program consisted of 14 messages providing key information about LCS (eg, eligibility, benefits, and harms) and a link to the DA for more detailed information. This program emphasized the importance of understanding both the benefits and harms of LCS, SDM, and smoking cessation and encouraged patients to discuss LCS with their provider at their next visit (Table 1). We delivered 1 text message per day for 14 days, always in the same order.

**Table 1.** Example of lung cancer screening text messages and learning objectives.

Learning objective of each text message	Text message
Introduction	Hi [FIRST NAME], this is the Lung Cancer Screening Message Center at xxxx. Your appointment with your primary care doctor is coming up soon. Based on your smoking history, we would like to send you text messages about lung cancer screening for the next 14 days. We would like you to learn about lung cancer screening before your next visit.
Pros and cons of lung cancer screening	Lung cancer screening has both pros and cons. It is important to learn about the pros and cons before you decide whether to have lung cancer screening.
What are cons of lung cancer screening?	What are the cons of lung cancer screening? One con is that you could get a false alarm. Lung cancer screening may find spots in the lungs that are NOT due to cancer. If this happens, you could need more testing to find out if the spots are due to cancer or NOT due to cancer.
Your value and preference	Should you be screened? Talk with your doctor to decide whether lung cancer screening is right for you. It will depend on your overall health and what is most important to you. Refer to the pamphlet that we sent to you if you would like to learn more. [a link to a decision aid].
Smoking cessation	Lung cancer screening does not replace the need for quitting smoking. As you know, quitting smoking could reduce your chance of getting lung cancer.
Closing message	We hope that you feel ready to talk about lung cancer screening with your doctor at your next visit. Think about some questions that you would like to ask. You can go to the website: [a link to a decision aid] for more information. We hope you found these messages helpful!

### Study Protocol

After obtaining informed consent, the study staff administered a baseline survey over the phone. Then, the study staff mailed the paper-based DA to all participants and started a text messaging intervention for the participants in the DA+TM group. The study staff confirmed the completion of the PCP visit in the EHR and aimed to conduct a follow-up telephone survey within 2 business days of the index primary care visit. The EHR was reviewed 3 and 6 months after the index visit for the completion of LDCT for LCS and regular chest computed tomography (CT).

We chose a sequential quasi-experimental design so that we could incrementally learn the process of the primary care flow, monitor the timing of recruitment, interventions, and PCP visits, and revise our study protocol if necessary. We originally planned to enroll the first 30 participants in the DA group and the next 30 participants in the DA+TM group. Due to the COVID-19 pandemic, recruitment was slower than expected. Thus, after we enrolled the first 16 participants in the DA group, we switched our enrollment efforts from the DA group to the DA+TM group. After enrolling 30 participants in the DA+TM group, we switched back to the DA group and enrolled 3 additional participants. After enrolling a total of 49 participants, we ended recruitment since we ran out of the recruitment pool from the 6 primary care clinics.

### Measures

#### Recruitment and Retention

We monitored the rates of recruitment and retention from study enrollment to the completion of follow-up surveys. We also monitored whether participants kept the original PCP appointments or rescheduled their appointments using the EHR.

#### Participant Characteristics

During recruitment calls, we collected data on participants’ ages and detailed smoking history to confirm study eligibility. During the baseline survey call, we collected data on the remaining demographics, family history of lung cancer, previous technology use, and perceived lung cancer risk [17]. We measured reading health literacy with the Single-Item Literacy Screener and listening health literacy with the item developed by the Health Literacy Cancer Prevention research team (Table S1 in Multimedia Appendix 1) [18,19].

#### Patient Engagement in DA and Text Messages

To evaluate intervention feasibility, we measured patient engagement with the mailed DA and text messages by self-report. We asked how much of these materials participants read and whether they clicked the link to a DA in the text messages in a follow-up survey (Table S1 in Multimedia Appendix 1).

#### LCS Discussion and SDM

Patient self-reported LCS discussion and SDM were measured at follow-up after the PCP visit. We asked participants whether they talked about LCS with their providers. If they had an LCS discussion, we asked participants who initiated the LCS discussion, whether their providers made LCS recommendations, and what recommendations they made (Table S1 in Multimedia Appendix 1).

The degree of SDM was measured with the shared decision-making process survey that consists of 4 items [20]. Participants were asked how much they discussed with their providers about the reason to have or not to have LCS (recorded as A lot or Some=1, A little or Not at all=0), whether their provider explained the options (to have or not to have LCS or to take more time to decide), and whether their providers asked the participant’s desire to proceed with LCS (recorded as Yes=1, or No=0). The total SDM process score ranges from 0 to 4, and a higher score indicates more SDM.

#### LCS Knowledge

Participants’ LCS knowledge, such as benefits and harms of LCS, was measured at baseline and follow-up using 9 questions adapted from a previous study (Table S2 in Multimedia Appendix 1) [10]. We created the LCS knowledge score, with the number of correct responses ranging from 0 to 9.

#### Decisional Conflict

Decisional conflict was measured with the 10-item Decisional Conflict Scale (DCS) in baseline and follow-up surveys [21,22]. The DCS score ranges from 0 (no conflict) to 100 (high conflict), and a score lower than 25 is associated with implementing a decision.

#### Stage of Decision-Making

The stage of decision-making was assessed with the “Stage of Decision Making” tool at baseline and in follow-up surveys [23]. The stage of decision-making is the individual’s readiness to engage in decision-making, progress in making a choice, and willingness to consider or reconsider options. We measured the stage of decision-making because the individual may respond differently to decision support or need different decision support depending on their stage of decision-making.

#### LCS Uptake

LDCT order, appointment, and completion data were collected from the EHR at 3 and 6 months after the PCP visit. We also measured regular chest CT completion at 6 months after the PCP visit.

### Statistical Analysis

Since this is a pragmatic trial in a real-world primary care setting, we included all participants regardless of whether they received text messages, rescheduled their appointments, or had an LCS discussion with their providers during their visits, and conducted an intention-to-treat analysis. Descriptive statistics were computed for participants’ demographics, responses to surveys, and LDCT outcomes. Continuous variables were summarized as median and IQR. Categorical variables were summarized as frequencies and proportions. The proportions of smoking status, health literacy, and the baseline stage of decision-making were compared by Fisher exact test. We first compared the LCS knowledge and DCS scores between baseline and follow-up surveys in each group using the Wilcoxon rank-sum test. Then, we compared the changes in LCS knowledge and DCS scores from baseline to follow-up surveys and SDM process scores between the groups by the Wilcoxon rank-sum test, and LCS discussion and LCS uptake between the arms by Fisher exact test. In addition, we conducted sensitivity analyses of SDM, LCS knowledge, and DCS scores among participants who reported LCS discussions with their providers. All statistical analyses were conducted using Stata 18 (StataCorp LLC).

### Ethical Considerations

This study was approved by the University of Massachusetts Chan Medical School institutional review board. Consent forms were mailed to all interested potential participants. A waiver of documentation of consent was obtained from the Institutional Review Board; all participants provided verbal informed consent before participation. Participants were offered a US $25 gift card for completion of each study survey (baseline and follow-up). Study data were handled in pseudonymized form using a coded key, accessible only to the research team. All personal data were stored securely on encrypted University of Massachusetts Chan Medical School servers. No identifiable images or personal information are included in the paper or any supplemental materials.

## Results

### Recruitment and Retention

**Figure 1.**
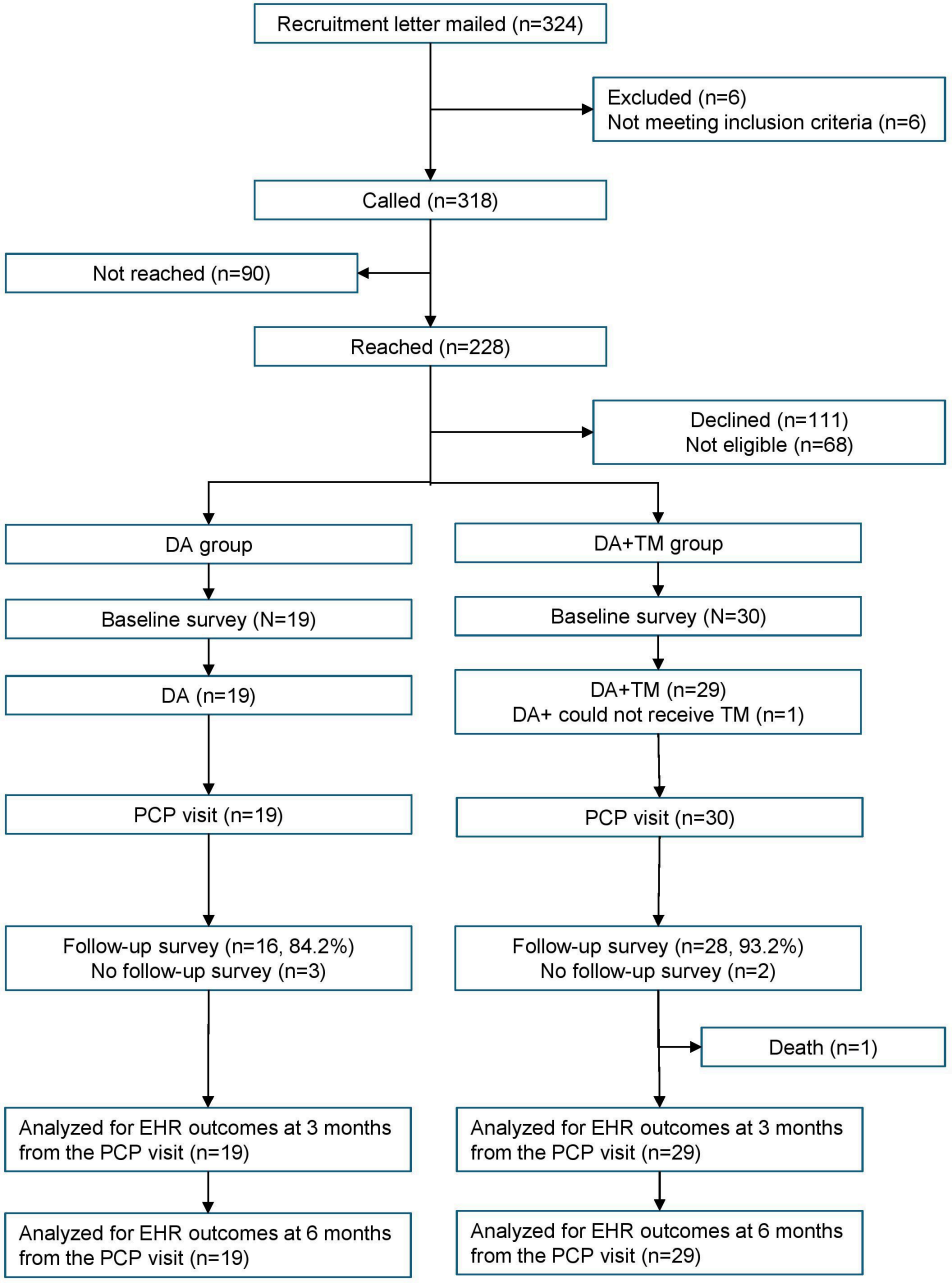
CONSORT (Consolidated Standards of Reporting Trials) flowchart. DA: decision aid; EHR: electronic health record; PCP: primary care provider; TM: text message.

The recruitment pool of 324 patients yielded 49 participants (19 in the DA group and 30 in the DA+TM group) with a recruitment rate of 15.4% (Figure 1). One participant in the DA+TM group could not receive text messages due to discontinuation of their text messaging plan. All participants had PCP visits. Notably, 4 (21%) participants in the DA group and 8 (27%) participants in the DA+TM group rescheduled their appointments. A total of 2 (11%) participants in the DA group had their appointment more than 4 weeks after the baseline survey. In the DA+TM group, 3 (10%) participants had their appointment within 2 weeks of the baseline survey, and 4 (13% ) participants had their appointment more than 5 weeks after the baseline survey. Overall, 16/19 (84%) participants in the DA group and 28/30 (93%) participants in the DA+TM group completed the follow-up survey. Due to appointment changes and participants’ availability, 2/16 participants in the DA group and 4/28 participants in the DA+TM group completed a follow-up survey more than 7 days after the PCP visit. For LCS uptake, that is, LDCT order and completion, we excluded 1 participant in the DA + TM group who passed away within 3 months after the PCP visit and included 19 participants in the DA group and 29 participants in the DA + TM group.

### Study Population

Table 2 describes participant characteristics. Participants were predominantly White, with 58% (28/48) female and a median age of 61 (IQR 57‐65) years. In terms of smoking status, 47% (9/19) of the participants in the DA group and 69% (20/29) of the participants in the DA+TM group reported currently smoking (*P*=.23). Regarding health literacy, 16% (3/19) of the participants in the DA group and 31% (9/29) of the participants in the DA+TM group reported limited reading health literacy (*P*=.32), and 53% (10/19) of the participants in the DA group and 76% (22/29) of the participants in the DA+TM group reported limited listening literacy (*P*=.12).

**Table 2.** Participant characteristics.

Characteristics	DA^a^ group (n=19)	DA+TM^b^ group (n=29)
Age in years, median (IQR)	60 (57-64)	62 (56-68)
Sex, n (%)		
Female	13 (68)	15 (52)
Male	6 (32)	14 (48)
Race, n (%)		
White	18 (95)	27 (93)
Black	0 (0)	0 (0)
Native American or Alaska Native	0 (0)	1 (3)
Other	1 (5)	1 (3)
Ethnicity, n (%)		
Hispanic or Latino	1 (5)	2 (7)
Annual income (US $), n (%)		
<30,000	8 (42)	12 (41)
30,000-59,999	6 (32)	9 (31)
60,000-99,999	3 (16)	2 (7)
>100,000	0 (0)	0 (0)
Prefer not to answer	2 (11)	6 (21)
Education level, n (%)		
High school graduate or less	6 (32)	13 (45)
Some college	8 (42)	8 (28)
Associate degree	3 (16)	1 (3)
Bachelor’s degree	2 (11)	6 (21)
Advanced college degree	0 (0)	1 (3)
Limited reading health literacy, n (%)	3 (16)	9 (31)
Limited listening health literacy, n (%)	10 (53)	22 (76)
Technology use, n (%)		
Smartphone ownership	18 (95)	23 (79)
Regular email usage	14 (74)	19 (66)
Text message usage	18 (95)	27 (93)
Previous patient portal usage	14 (74)	21 (72)
Smoking status, n (%)		
Current	9 (47)	20 (69)
Smoking duration by pack-year, median (IQR)	44 (33-52.5)	52 (33.5-52)
General health, n (%)		
Excellent	3 (16)	1 (3)
Very good	1 (5)	4 (14)
Good	7 (37)	9 (31)
Fair	6 (32)	12 (41)
Poor	2 (11)	3 (10)
Mental health, n (%)		
Excellent	3 (16)	5 (17)
Very good	4 (21)	5 (17)
Good	8 (42)	9 (31)
Fair	3 (16)	6 (21)
Poor	1 (5)	4 (14)
Family history of lung cancer, n (%)	10 (53)	13 (45)
Perceived lung cancer risk^c^, n (%)		
Very low	3 (16)	5 (18)
Somewhat low	5 (26)	2 (7)
Moderate	5 (26)	10 (36)
Somewhat high	4 (21)	9 (32)
Very high	2 (11)	2 (7)

a DA: decision aid.

b TM: text message.

c n=1 missing data in the DA+TM group.

### DA and Text Message Engagement

In the DA group, 63% (10/16) of the participants reported reading all or most of the DA while in the DA+TM group, 57% (16/28) of the participants reported reading all or most of the DA, and 21% (6/28) of the participants reported reading none of it (Table 3). In the DA+TM group, 75% (21/28) of the participants reported reading all or most of the text messages, and only 39% (11/28) of the participants reported using the link to access the DA. Among the 6 participants who did not read a mailed DA at all in the DA+TM group, none accessed DA from the links provided in the text messages. However, 5 participants read all or most of the text messages, while 1 participant read only a few of the text messages. In summary, 63% (10/16) of the participants in the DA group reported reading all or most of the mailed DA and 82% (23/28) of the participants in the DA+TX group reported reading all or most of the mailed DA or the text messages (*P*=.17).

**Table 3.** Patient self-report engagement in the intervention.

DA^a^ and TM^b^ engagement	DA group (n=16)	DA+TM group (n=28)
Decision-aid reading, n (%)		
All or most	10 (63)	16 (57)
Some or a little	6 (38)	4 (14)
None	0 (0)	6 (21)
Did not receive, do not remember	0 (0)	2 (7)
Text message reading, n (%)		
All or most	—^c^	21 (75)
Some or a few	—	6 (21)
None	—	0 (0)
Could not receive text messages	—	1 (4)

a DA: decision aid.

b TM: text message.

c Not applicable.

### LCS Discussion and SDM

Most participants (94%, 15/16 in the DA group and 82%, 23/28 in the DA+TM group) reported having an LCS conversation with their PCPs during the visit (*P*=.39). Furthermore, 56% (9/16) of the participants in the DA group and 57% (16/28) of the participants in the DA+TM group stated they initiated the LCS conversation (*P*=1.00). In terms of the provider’s LCS recommendation, 63% (10/16) of the participants in the DA group and 61% (17/28) of the participants in the DA+TM group reported receiving recommendations from their providers (*P*=1.00). With the exception of 1 participant in the DA+TM group who was advised not to undergo LCS, 26/27 participants in the 2 groups were recommended for LCS.

The median of the SDM process score measuring degree of SDM (with 0 being low and 4 being high) was 3.0 (IQR 1.5‐4.0) in the DA group (n=16) and 2.0 (IQR 1.0‐3.0) in the DA+TM group (n=26 due to 2 missing data; *P*=.11).

### LCS Knowledge

**Figure 2.**
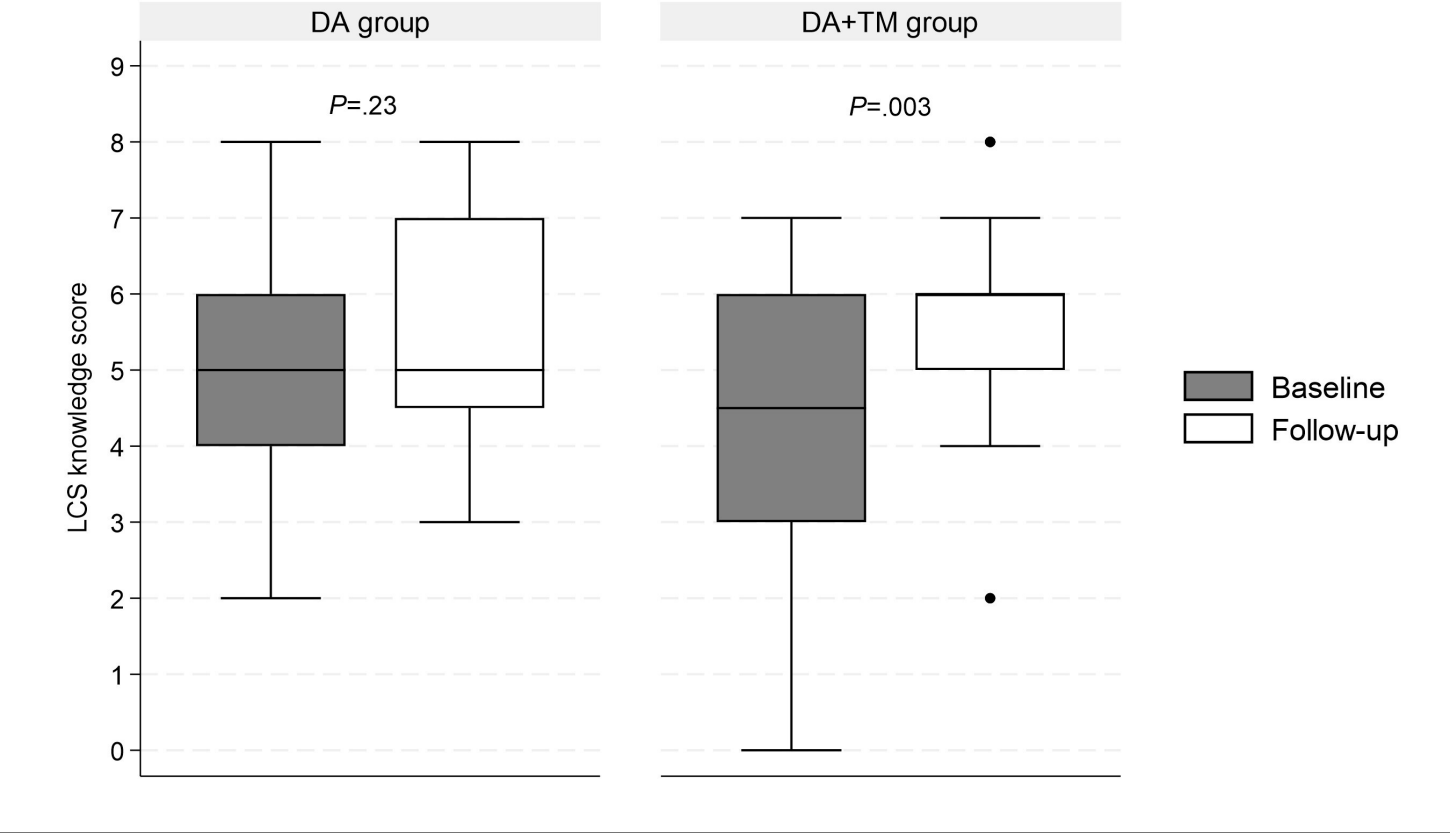
Lung cancer screening knowledge at the baseline and follow-up surveys. Boxes indicate 25th to 75th percentile. Dots indicate an outlier. DA: decision aid; LCS: lung cancer screening; TM: text messages. Lines indicate a median.

The median patient LCS knowledge score in the DA group was 5 of 9 (IQR 4‐6) at baseline and 5 of 9 (IQR, 4.5‐7) at follow-up, without significant change (*P*=.23). On the other hand, the median patient LCS knowledge in the DA+TM group significantly improved from 4.5 (IQR 3‐6) at baseline to 6 (IQR 5‐6) at follow-up (*P*=.003; Figure 2). However, there was no statistically significant difference in the change of LCS knowledge score from baseline to follow-up between the 2 groups (DA: 0.5, IQR –1 to 2.5) and DA+TM: 1.5, IQR 0‐3; *P*=.24). Details about performance by group on specific LCS knowledge questions are provided in Table S2 in Multimedia Appendix 1.

### Decisional Conflict

**Figure 3.**
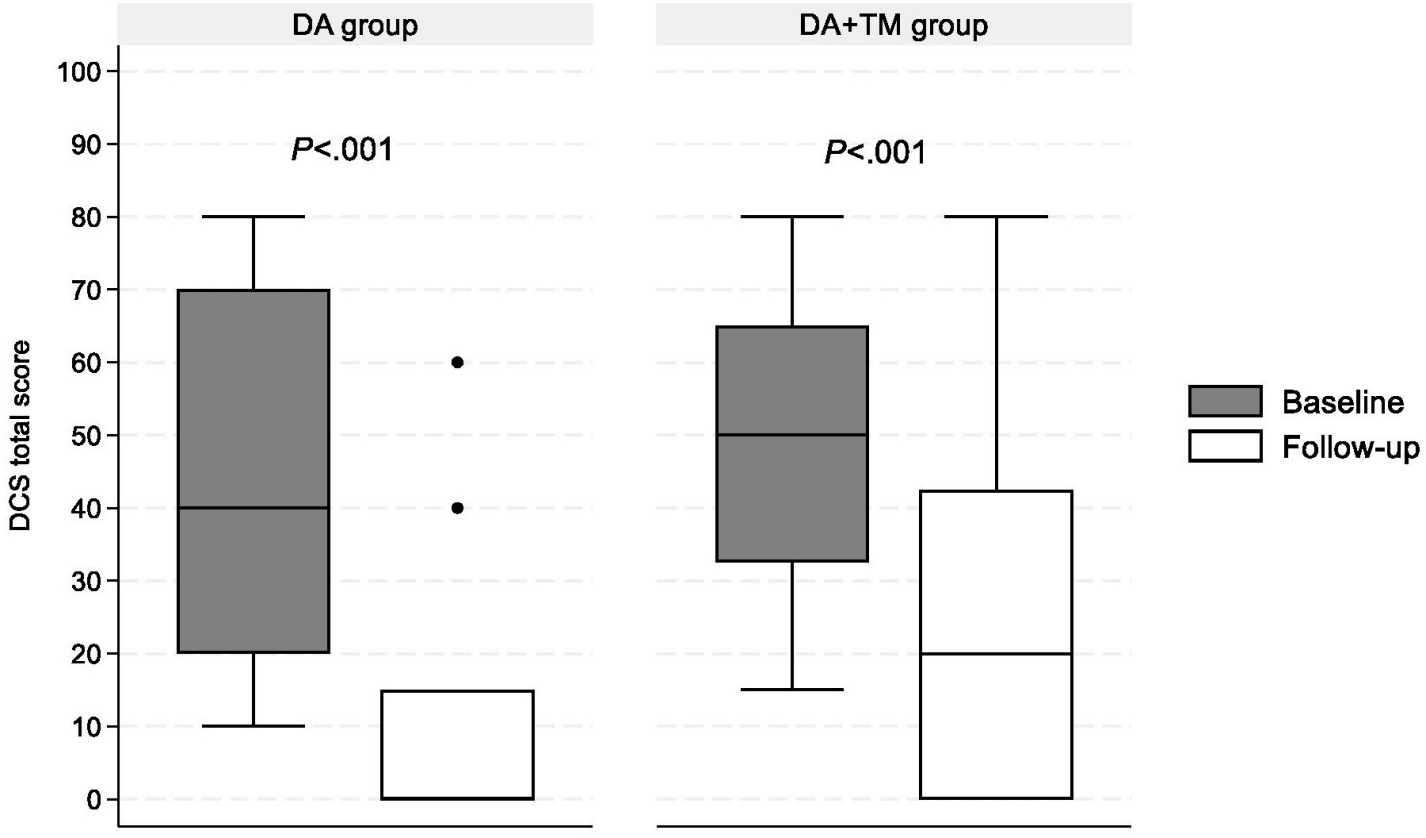
Decisional conflict scale at the baseline and follow-up surveys. Lines indicate a median. Boxes indicate 25th to 75th percentile. Dots indicate an outlier. DA: decision aid, DCS: decisional conflict scale; TM: text messages.

The total median DCS decreased significantly from 40 (IQR 20.0‐70.0) at baseline to 0 (IQR 0‐15.0) at follow-up in the DA group (n=15 with 1 missing data; *P*<.001) and from 50.0 (IQR 32.5‐65.0) to 20 (IQR 0‐42.5) in the DA+TM group (*P*<.001; Figure 3). The median change of the total DCS was 25.0 (IQR 15.0‐50.0) in the DA group and 20.0 (IQR 10.0‐47.5) in the DA+TM group (*P*=.50). A DCS of less than 25 means the participants were ready to make a decision. At baseline, 31.3% (5/16) of the participants in the DA group and 14.3% (4/18) of the participants in the DA+TM group had a total DCS less than 25 (*P*=.25). At follow-up, 80% (12/15, n=1 missing data) of the participants in the DA group and 50% (14/28) of the participants in the DA+TM group had the total DCS less than 25 (*P*=.10). Figures S1 and Table S3 in Multimedia Appendix 1 describe the Informed, Value, Support, and Uncertainty subscales.

### Stage of Decision-Making

**Figure 4.**
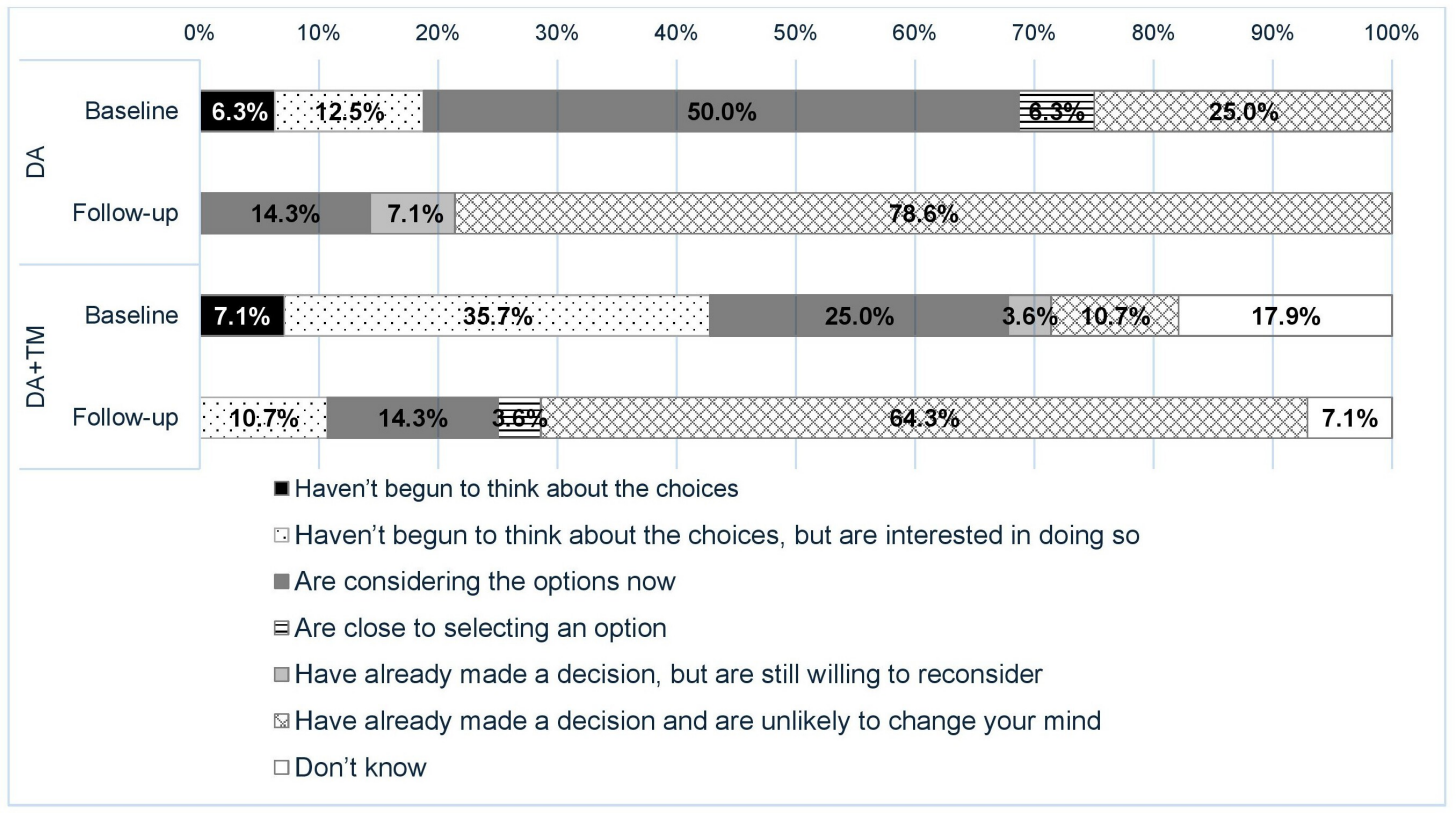
Stage of decision making at the baseline and follow-up surveys. DA: decision aid, TM: text messages.

At baseline, the participants were at diverse stages of decision-making about LCS. Among the participants in the DA group, 19% (3/16) of the participants had not begun to think about the choices, 50% (8/16) of the participants were considering the options, and 25% (4/16) of the participants had already made a decision and were unlikely to change their minds (Figure 4). Among the participants in the DA+TM group, 43% (12/28) of the participants had not begun to think about the choices, 25% (7/28) of the participants were considering the options, and 11% (3/28) of the participants had already made a decision and were unlikely to change their minds. There was no statistically significant difference in the baseline stages of decision-making between the 2 groups (*P*=.07). The stage of decision-making shifted toward decision-making at follow-up in both groups, and 79% (11/14, 2 missing data) of the participants in the DM group and 64% (18/28) of the participants in the DA+TM group stated that they had already made a decision and were unlikely to change their mind.

### LCS Uptake

Table 4 describes LDCT ordering, scheduling, and completion rates at 3 and 6 months after the index PCP visit. Within 3 months after the index PCP visit, 26% (5/19) of the participants in the DA group and 48% (14/29) of the participants in the DA+TM group had LDCT orders (*P*=.15), and 5% (1/19) of the participants in the DA group and 31% (9/29) of the participants in the DA+TM group completed LDCT for LCS (*P*=.07). Within 6 months after the index PCP visit, 26% (5/19) of the participants in the DA group and 34% (10/29) of the participants in the DA+TM group completed LDCT for LCS (*P*=.75). Also, 2 participants in each group had chest CT for other indications during the study period.

**Table 4.** Lung cancer screening uptake.

LDCT^a^ for LCS^b^	DA^c^ group (n=19)	DA+TM^d^ group (n=29)	*P* value
3 months after PCP^e^ visit, n (%)			
Order	5 (26)	14 (48)	.15
Appointment	3 (16)	12 (41)	.11
Completion	1 (5)	9 (31)	.07
6 months after PCP visit, n (%)			
Order	7 (37)	14 (48)	.56
Appointment	7 (37)	13 (45)	.77
Completion	5 (26)	10 (34)	.75
Chest CT^f^, n (%)			
6 months from PCP visit	2 (11)	2 (7)	1.00

a LDCT: low-dose computed tomography.

b LCS: lung cancer screening.

c DA: decision aid.

d TM: text message.

e PCP: primary care provider.

f CT: computed tomography.

### Sensitivity Analysis of the Participants Who Reported LCS Discussion

Tables S4-S6 and Figures S2 and S3 in Multimedia Appendix 1 describe the results of the sensitivity analysis of SDM, LCS knowledge, decision conflict, and LCS uptake among participants who reported LCS discussion during their PCP visit. Our findings of SDM, LCS knowledge, decisional conflict, and LCS uptake were similar between our sensitivity and primary analyses among all participants.

## Discussion

### Principal Findings

We successfully conducted a sequential quasi-experimental pilot study to evaluate the feasibility and to explore potential outcomes of the previsit preparation strategies for SDM in LCS in primary care settings. Our study protocol appeared to be feasible, given that our recruitment rate was similar to that of other studies evaluating the effectiveness of a DA for LCS in primary care settings [24,25] and the retention rate was high. We found that previsit preparation using a mailed DA with or without automated text messaging was feasible. Most participants in both groups reported discussing LCS with their provider. Patient decision conflict was significantly reduced in both groups from baseline to follow-up. We observed significant improvement in LCS knowledge among the participants in the DA+TM group, but not among those in the DA group. These findings provide promising preliminary signals on the potential of the enhanced previsit preparation using text messaging to broadly reach patients eligible for LCS and to improve informed decision-making and uptake of LCS. However, the degree of SDM remained limited in both groups.

LCS discussion rates in our study exceeded 80% in both groups, surpassing the 60% rates reported in a previous study about SDM in LCS [25]. Furthermore, approximately 60% of the participants in our study reported initiating the LCS conversation. Although the exact reason for the high LCS discussion rates is unclear, receiving the information in advance rather than immediately before their PCP visit might have provided more time for patients to think about and prepare for LCS discussions with their providers.

In our pilot study, the 6 participants who did not read DAs reported reading text messages. This finding supports the high acceptability of text messages observed during the development of the LCS text messages and aligns with prior studies showing similar acceptability for text messages to support SDM for colon cancer screening among individuals diverse in income, race, and health literacy [26]. Individuals with low income and limited health literacy often need assistance to review DAs [10]. Thus, text messages have the potential to improve the reach and engagement of broader LCS-eligible populations in SDM.

In the DA+TM group, patient LCS knowledge significantly improved at follow-up, despite 31% reporting limited health literacy. Presenting new information in small doses in a sequential manner may have decreased cognitive load for learners, especially for those with limited health literacy [27,28]. However, the participants in both groups still had a deficit of detailed knowledge on the potential harms of LCS and the limited degree of SDM, which may reflect more on providers’ practice than patients’ factors. Improving patient understanding and SDM may require a provider-level intervention in addition to this patient-facing previsit intervention.

### Comparison With Prior Work

Despite the CMS mandate, how best to reach and support patients eligible for LCS in SDM in primary care settings is unknown. Various DAs for LCS have been shown to improve patient knowledge and decrease decisional conflict in study settings [9]. Web-based DAs increased the uptake of LCS in primary care settings but did not improve the quality of SDM in previous randomized controlled trials (RCTs) [24,25]. Still, adoption of DAs in real-world clinical settings remains challenging [29].

Our pilot study differed from previous studies in 2 main ways [24,25]. First, we used an automated text messaging system as a channel to reach and deliver information to patients. Second, we delivered a DA and text messaging program to the participants outside of clinical and study visits, whereas previous RCTs delivered DAs during the study visit right before the primary care visit [24,25]. This approach is potentially more feasible in real-world clinical settings with limited resources.

### Limitations

This study has some limitations. First, our participants were predominantly White and from a single health care system, limiting generalizability. However, our participants were diverse in socioeconomic status. Second, the sample size of this pilot study was small and there was an imbalance between the 2 groups. Third, there may have been differences in participants’ characteristics and stages of decision-making between the 2 groups that could have affected the outcomes. In addition, the temporal effect of the COVID-19 pandemic and unmeasured providers’ characteristics and SDM practices may have affected the outcomes due to the sequential design of the study. Thus, comparability and statistical power for between-group analyses of participants’ characteristics and outcomes are limited. A RCT with a larger sample size powered to detect clinically meaningful differences between groups is needed for further evaluation. Still, our pilot study demonstrated interesting preliminary findings to explore how to help patients prepare for LCS discussions prior to their primary care visits.

### Conclusions

An enhanced previsit preparation strategy using an automated text messaging program is feasible in primary care settings and potentially effective in reaching and engaging the broader LCS-eligible population in SDM for LCS. Our pilot study suggests that a previsit automated text messaging program combined with a mailed DA may improve basic LCS knowledge without exacerbating disparities related to the digital divide or health literacy level. Previsit preparation enhanced by a text messaging program may facilitate SDM discussions about LCS and ultimately increase LCS uptake.

## Supplementary material

10.2196/69044 Multimedia Appendix 1 Additional materials.

10.2196/69044 Checklist 1 CONSORT checklist.

## Abbreviations

CMS: Centers for Medicare & Medicaid Services
CONSORT: Consolidated Standards of Reporting Trials
CT: computed tomography
DA: decision aid
DCS: Decisional Conflict Scale
EHR: electronic health record
LCS: lung cancer screening
LDCT: low‐dose computed tomography
PCP: primary care provider
RCT: randomized controlled trial
SDM: shared decision-making
