# Supplementary material for: Previsit Preparation for Shared Decision-Making in Lung Cancer Screening in Primary Care Using a Paper Decision Aid and an Automated Text Messaging Program: Quasi-Experimental Pilot Study
Source: JMIR Form Res. 2025 Sep 17;9:e69044. doi: 10.2196/69044 (PMC12443356; doi:10.2196/69044)
Supplement: Multimedia Appendix 1 [file formative-v9-e69044-s001.docx]

**Table 1. Measure**

| **Construct** | **Questions and Responses** |
| --- | --- |
| Perceived lung cancer risk | “How likely do you think it is that you will develop lung cancer in the future?”  1-Very low, 2-Somewhat low, 3-Moderate, 4-Somewhat high, 5-Very high |
| Reading health literacy | “How often do you need to have someone help you when you read instructions, pamphlets, or other written material from your doctor or pharmacy?”  1-Never, 2-Rarely, 3-Sometimes, 4-Often, 5-Always  Scores >2: positive for limited reading health literacy |
| Listening health literacy | “I have a hard time understanding when people speak quickly.”  1- Strongly agree, 2-Agree, 3-Disagree, 4-Strongly disagree  Score 1 or 2: positive for limited listening health literacy |
| Patient engagement in a mailed decision aid | “How much of the decision aid did you read prior to your visit?”  1-All, 2-Most, 3-Some, 4-A little, 5-None |
| Patient engagement in text messages | “How many of the text messages did you read?”  1-All, 2-Most, 3-Some, 4-A little, 5-None |
| Patient and provider LCS discussion | “Did you and [provider’s name] talk about lung cancer screening during the visit?”  1-Yes, 2-No, 3-Not sure/Don’t remember |
| The person who initiated the LCS discussion | “Who brought up the topic of LCS during your visit?”  1-Patient, 2-Provider, 3-Don’t remember |
| A provider’s LCS recommendation | “Did [provider’s name] make any recommendation as to whether you should or should not have lung cancer screening?”  1-Yes, 2-No, 3-Not sure/don’t remember  “What did [provider’s name] recommend?”  1-I should have LCS, 2-I should not have LCS, or 3-I should decide for myself whether to have LCS, 4-Other |

LCS, lung cancer screening

**Table 2. LCS knowledge question responses at the baseline and follow-up surveys**

|  | | | **DA group (n=16)** | | **DA+TM group (n=28)** | |
| --- | --- | --- | --- | --- | --- | --- |
|  | | | **Baseline** | **Follow-up** | **Baseline** | **Follow-up** |
| **LCS knowledge, n (%)** | | |  |  |  |  |
|  | 1. Getting screened for lung cancer involves having a chest CT scan at least every year. | |  |  |  |  |
|  |  | True^a^ | 8 (50.0) | 5 (31.3) | 5 (17.9) | 19 (67.9) |
|  |  | False | 0 (0) | 4 (25.0) | 3 (10.7) | 2 (7.1) |
|  |  | I don’t know/Unsure | 8 (50.0) | 7 (43.8) | 20 (71.4) | 7 (25.0) |
|  | 2. All smokers should be screened for lung cancer. | |  |  |  |  |
|  |  | True | 10 (62.5) | 9 (56.3) | 19 (67.9) | 21 (75.0) |
|  |  | False^a^ | 1 (6.3) | 6 (37.5) | 2 (7.1) | 3 (10.7) |
|  |  | I don’t know/Unsure | 5 (31.3) | 1 (6.3) | 7 (25.0) | 4 (14.3) |
|  | 3. Lung cancer screening lowers your chances of getting lung cancer. | |  |  |  |  |
|  |  | True | 5 (31.3) | 4 (25.0) | 1 (3.6) | 8 (28.6) |
|  |  | False^a^ | 9 (56.3) | 10 (62.5) | 23 (82.1) | 19 (67.9) |
|  |  | I don’t know/Unsure | 2 (12.5) | 2 (12.5) | 4 (14.3) | 1 (3.6) |
|  | 4. Lung cancer screening can cure cancer. | |  |  |  |  |
|  |  | True | 2 (12.5) | 1 (6.3) | 0 | 0 |
|  |  | False^a^ | 14 (87.5) | 14 (87.5) | 22 (78.6) | 27 (96.4) |
|  |  | I don’t know/Unsure | 0 | 1 (6.3) | 6 (21.4) | 1 (3.6) |
|  | 5. Lung cancer screening lowers your chances of dying from lung cancer. | |  |  |  |  |
|  |  | True^a^ | 11 (68.8) | 10 (62.5) | 19 (67.9) | 20 (71.4) |
|  |  | False | 2 (12.5) | 5 (31.3) | 3 (10.7) | 7 (25.0) |
|  |  | I don’t know/Unsure | 3 (18.8) | 1 (6.3) | 6 (21.4) | 1 (3.6) |
|  | 6. Lung cancer screening lowers your chances of developing lung nodules. | |  |  |  |  |
|  |  | True | 5 (31.3) | 4 (25.0) | 8 (28.6) | 5 (17.9) |
|  |  | False^a^ | 8 (50.0) | 10 (62.5) | 11 (39.3) | 16 (57.1) |
|  |  | I don’t know/Unsure | 3 (18.8) | 2 (12.5) | 9 (32.1) | 7 (25.0) |
|  | 7. You may find some things in your lungs that are not cancer but would need an extra test to check. | |  |  |  |  |
|  |  | True^a^ | 16 (100) | 15 (93.8) | 23 (82.1) | 27 (96.4) |
|  |  | False | 0 | 0 | 0 | 0 |
|  |  | I don’t know/Unsure | 0 | 1 (6.3) | 5 (17.9) | 1 (3.6) |
|  | 8. You may need to get an extra test which can cause complications. | |  |  |  |  |
|  |  | True^a^ | 8 (50.0) | 11 (68.8) | 7 (25.0) | 13 (46.4) |
|  |  | False | 0 | 1 (6.3) | 6 (21.4) | 3 (10.7) |
|  |  | I don’t know/Unsure | 8 (50.0) | 4 (25.0) | 15 (53.6) | 12 (42.9) |
|  | 9. There are no harms associated with screening. | |  |  |  |  |
|  |  | True | 4 (25.0) | 4 (25.0) | 16 (57.1) | 7 (25.0) |
|  |  | False^a^ | 5 (31.3) | 10 (62.5) | 4 (14.3) | 12 (42.9) |
|  |  | I don’t know/Unsure | 7 (43.8) | 2 (12.5) | 8 (28.6) | 9 (32.1) |

^a^ Indicates correct answers, DA: decision aid, TM: text messages

**Figure 1. Decisional conflict score subscales at baseline and follow-up surveys**

|  | **DA group (n=16)** | |  | **DA+TM group (n=28)** | |  |
| --- | --- | --- | --- | --- | --- | --- |
|  | **Baseline** | **Follow-up** | ***P* value** | **Baseline** | **Follow-up** | ***P* value** |
| **Scale, median (IQR**) |  |  |  |  |  |  |
| **Informed subscale** | 66.7 (50-75) | 0 (0-8.3) | <.001 | 75.0 (50.0-100) | 25.0 (0-50.0) | <.001 |
| **Value clarity subscale** | 50.0 (0-87.5) | 0 (0-0) | .002 | 62.5 (12.5-100) | 12.5 (0-50.0) | <.001 |
| **Support subscale** | 16.7 (0-33.3) | 0 (0-0) | .05 | 33.3 (0-33.3) | 0 (0-25.0) | .03 |
| **Uncertainty subscale** | 0 (0-75.0) | 0 (0-25.0) ^a^ | .09 | 25.0 (0-62.5) | 0 (0-50.0) | .21 |

DA: decision aid, TM: text messages

Blue indicates baseline. Pink indicates follow-up. Lines indicate a median. Boxes indicate 25^th^ to 75^th^ percentile. Dots indicate an outlier.

**^a^**: 1 missing data. Abbreviation: IQR, interquartile range

**Table 3. Change of decisional conflict scale scores from baseline to follow-up**

|  | **DA group (n=16)** | **DA+TM group (n=28)** | ***P* value** |
| --- | --- | --- | --- |
| **Change of scale,**  **median (IQR**) |  |  |  |
| **Informed subscale** | 58.3 (41.7-66.7) | 50.0 (0-75.0) | .39 |
| **Value clarity subscale** | 37.5 (0-62.5) | 25.0 (0-50.0) | .61 |
| **Support subscale** | 0 (0-33.3) | 8.3 (0-33.3) | .99 |
| **Uncertainty subscale** | 0 (0-25.0) ^a^ | 0 (0-37.5) | .72 |
| **Total DCS** | 25.0 (15.0-50.0) ^a^ | 20.0 (10.0-47.5) | .50 |

**^a^**: 1 missing data

DA: decision aid, TM: text messages

**Table 4. SDM process 4 score among the participants with LCS discussion**

|  | **DA group (n=15)** | **DA+TM group (n=23)** | ***P* value** |
| --- | --- | --- | --- |
| **SDM process 4, median (IQR)** | 3.0 (2.0-4.0) | 2.0 (1.0-3.0) | .16 |

IQR, interquartile range

DA: decision aid, TM: text messages

**Figure 2. LCS knowledge among the participants with LCS discussion**

|  | **DA group (n=15)** | | | **DA+TM group (n=23)** | | |
| --- | --- | --- | --- | --- | --- | --- |
|  | **Baseline** | **Follow-up** | ***P* value** | **Baseline** | **Follow-up** | ***P* value** |
| **LCS knowledge score, median, (IQR)** | 5 (4-6) | 5 (4-7) | .10 | 4 (2-6) | 6 (5-7) | .002 |

|  | **DA group (n=15)** | **DA+TM group (n=23)** | ***P* value** |
| --- | --- | --- | --- |
| **LCS knowledge change,**  **median, (IQR)** | 1.0 (-1.0–3.0) | 2.0 (0-3.0) | .17 |

LCS: lung cancer screening, DA: decision aid, TM: text messages

Lines indicate a median. Boxes indicate 25^th^ to 75^th^ percentile. Dots indicate an outlier.

**Figure 3. Decisional conflict score among the participants with LCS discussion**

|  | **DA group (n=15)** | | | **DA+TM group (n=23)** | | |
| --- | --- | --- | --- | --- | --- | --- |
|  | **Baseline** | **Follow-up** | ***P* value** | **Baseline** | **Follow-up** | ***P* value** |
| **Scale, Median (IQR)** |  |  |  |  |  |  |
| DCS total scale | 35.0 (20.0-70.0) | 0 (0-5.0) | .001 | 50.0 (25.0-65.0) | 15.0 (0-40.0) | <.001 |
| Informed subscale | 66.7 (50.0-66.7) | 0 (0-0) | .001 | 66.7 (50.0-100) | 16.7 (0-33.3) | <.001 |
| Value clarity subscale | 50.0 (0-100.0) | 0 (0-0) | .003 | 50.0 (0-100) | 0 (0-50.0) | .003 |
| Support subscale | 0 (0-33.3) | 0 (0-0) | .01 | 33.3 (0-33.3) | 0 (0-16.7) | .02 |
| Uncertainty subscale | 0 (0-75.0) | 0 (0-25.0) | .08 | 25.0 (0-50.0) | 0 (0-50.0) | .16 |

DCS: decisional conflict scale, DA: decision aid, TM: text messages

Lines indicate a median. Boxes indicate 25^th^ to 75^th^ percentile. Dots indicate an outlier.

**Table 5. Change of decisional conflict scale score among the participants with LCS discussion**

|  | **DA group (n=15)** | **DA+TM group (n=23)** | ***P* value** |
| --- | --- | --- | --- |
| **Change of scale,**  **median (IQR**) |  |  |  |
| **Informed subscale** | 50.0 (33.3-66.7) | 50.0 (16.7 -83.3) | .73 |
| **Value clarity subscale** | 50.0 (0-75.0) | 25.0 (0-50.0) | .57 |
| **Support subscale** | 0 (0-33.3) | 16.7 (0-33.3) | .59 |
| **Uncertainty subscale** | 0 (0-25.0) | 0 (0-50.0) | .82 |
| **Total DCS** | 22.5 (15.0-40.0) | 25 (10.0-50.0) | .97 |

**Table 6. Lung cancer screening uptake among the participants with LCS discussion**

|  | **DA group (n=15)** | **DA+TM group (n=23)** | **P value** |
| --- | --- | --- | --- |
| **LDCT, n (%)** |  |  |  |
| **3 months after PCP visit** |  |  |  |
| Order | 5 (33.3) | 13 (56.5) | .19 |
| Appointment | 3 (20,0) | 11 (47.8) | .10 |
| Completion | 1 (6.7) | 8 (34.8) | .06 |
| **6 months after PCP visit** |  |  |  |
| Order | 6 (40,0) | 13 (56.5) | .51 |
| Appointment | 6 (40.0) | 12 (52.2) | .52 |
| Completion | 4 (26.7) | 9 (39.1) | .50 |
| **Chest CT, n (%)** |  |  |  |
| 6 months from PCP visit | 1 (6.7) | 2 (8.7) | 1.00 |
